# Renal Autotransplantation for Iatrogenic High-Grade Ureteric Stricture

**DOI:** 10.1155/2012/259527

**Published:** 2012-12-04

**Authors:** Jose Soto Soto, Michael Phillips, Joseph Cernigliaro, William Haley

**Affiliations:** ^1^Division of Pulmonary and Critical Care Medicine, Mayo Clinic, Jacksonville, FL 32224, USA; ^2^Department of Internal Medicine, Mayo Clinic, Jacksonville, FL 32224, USA; ^3^Division of Hypertension and Nephrology, Mayo Clinic, Jacksonville, FL 32224, USA; ^4^Department of Diagnostic Radiology, Mayo Clinic, Jacksonville, FL 32224, USA

## Abstract

A 47-year-old Hispanic woman developed a chronically obstructed left kidney, due to a long-segment ureteric stricture deemed not amenable to reimplantation, following left ovarian cyst excision in 2004. Therefore, a ureteral stent requiring exchange every 3 months was necessary, due to hydronephrosis, recurrent urosepsis, chronic pain, and a poor quality of life. Her medical history was complicated by hypertension, poorly controlled diabetes mellitus, and microalbuminuria, suggesting early diabetic nephropathy. A left nephrectomy was recommended. This was deferred, due to concern for progressive kidney failure associated with her comorbidities. A radionuclide Tc-99m MAG3 renal scan revealed differential perfusion as follows: 44% left kidney and 56% right kidney, with symmetrical uptake on the renogram phase and delayed excretion on the left, which were correctted following furosemide administration. A left ureteronephrectomy with autotransplantation of the left kidney and ureteroneocystostomy was performed in 2009. Since then, the patient has experienced no further complications or need for invasive procedures, with excellent diabetic control and stable renal function (eGFR > 60 mL/min/1.73 m^2^). This technique is seldom employed in the surgical management of complex ureteral injuries, but may be an alternative for appropriate cases.

## 1. Introduction

Long-term management of a high-grade ureteric stricture can be extremely complicated and can imply significant morbidity and mortality for the patient. We present a case of an iatrogenic high-grade left ureteric stricture that led to multiple hospitalizations for stent exchange and treatment of urosepsis in a diabetic patient that was resolved by renal autotransplantation.

## 2. Case Report

**Figure 1:**
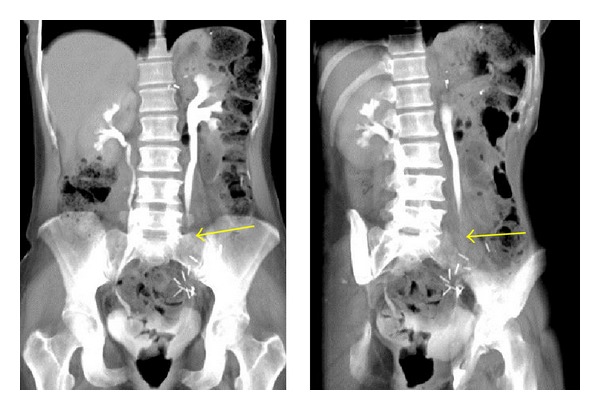
Left ureteral stricture.

**Figure 2:**
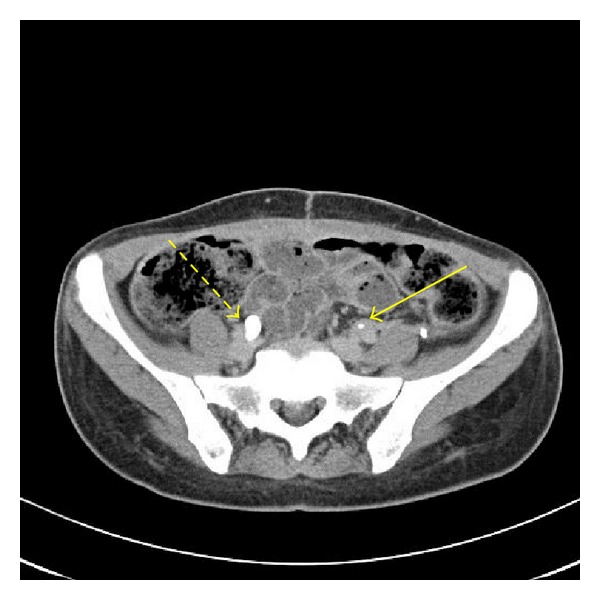
Left ureteral stricture (solid yellow arrow) and patent right ureter (interrupted yellow arrow).

A 47-year-old Hispanic woman developed chronic obstructive left kidney, due to high-grade left ureteric stricture, requiring multiple stent placements. Her medical history is significant for hypertension and insulin-dependent diabetes, status post total pancreatectomy, due to chronic pancreatitis. Originally, she underwent removal of a left ovarian cyst in 2004, which was then complicated by a left ureteral stricture (Figures 1 and 2). Since then, more than 12 ureteral stent replacement were required due to recurrent hydronephrosis and episodes of urosepsis.

Her renal function was normal (eGFR > 60%), with the exception of mild proteinuria suggestive of early diabetic nephropathy. Unfortunately, her diabetes was uncontrolled and she continued having left costovertebral angle tenderness and chronic hematuria and dysuria. Thus, the patient was felt to be at increased risk for infectious complications and left-sided nephrectomy was recommended. However, given her high risk for end-stage renal disease after nephrectomy, other options were explored.

**Figure 3:**
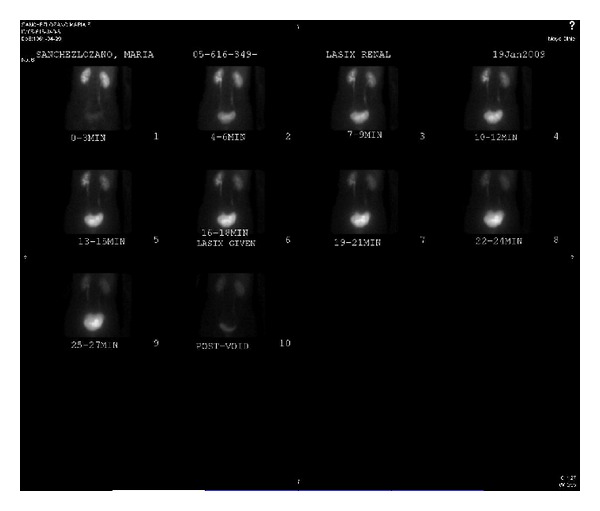
9.8 mCi Tc-99m MAG3 Radionuclide differential renal scan with furosemide. Blood flow phase of the study shows relatively symmetric perfusion; blood flow to the left kidney is minimally decreased with respect to the right kidney. Differential perfusion is 44% to the left kidney and 56% to the right kidney. Renogram phase of the study shows symmetric uptake bilaterally, with only minimally delayed excretion to 17 minutes in the left kidney, suggesting a mildly patulous collecting system. Following Lasix administration, there is normal clearance of activity bilaterally.

A radionuclide Tc-99m MAG3 differential renal scan with furosemide was obtained. Differential perfusion was 44% to the left kidney and 56% to the right kidney. Renogram phase of the study showed symmetric uptake bilaterally, with only minimally delayed excretion to 17 minutes in the left kidney, suggesting a mildly patulous collecting system. Following Lasix administration, there was normal clearance of activity bilaterally (Figure 3). It was decided that it was worth attempting to salvage her kidney, therefore a left kidney autotransplantation was suggested.

**Figure 4:**
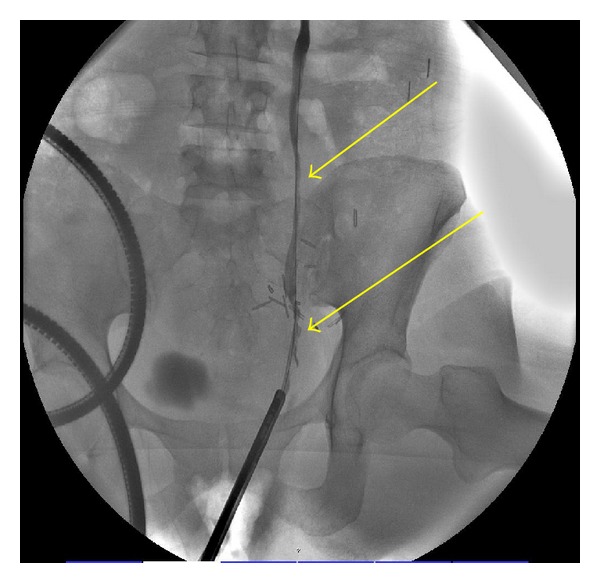
Retrograde pyelogram defines the exact location of the stricture (yellow arrows) and assesses for viable ureter to be used at the time of the renal auto transplantation.

Given her previous surgeries and significant retroperitoneal fibrosis, careful and extensive planning was required, including an MR angiogram of her left kidney as well as a retrograde pyelogram to define the exact location of the stricture and assess for viable ureter to be used at the time of the auto transplantation (Figure 4). A left ureteronephrectomy with autotransplantation of the left kidney to the right lower quadrant with ureteroneocystostomy and stent placement was performed successfully. Since then, the patient has had no further complications or need for invasive procedures and her renal function remains intact. 

Renal autotransplantation can be considered as a safe alternative method for the management of complex ureteric strictures.

## 3. Discussion

Renal autotransplantation was initially described in 1963 by Hardy and Eraslan, when they treated a high ureteric injury by reimplanting the repaired organ into the ipsilateral iliac fossa [1]. Currently, this technique is seldom used in the surgical management of complexureteral injuries,renal arteryaneurysms,renovascular hypertension, and renal malignancy [2]. Urinary tract injuries leading to urinary strictures are reported in approximately 1% of women who undergo pelvic surgery [3]. These are often recognized intraoperatively; otherwise, in a series of 20 urinary tract injuries in women after pelvic surgery, the mean time to diagnosis was 5.6 days (range 0 to 14 days) [4]. Common postoperative symptoms are leakage of urine from the vagina or abdominal incision, unilateral or bilateral flank pain, hematuria, oliguria, anuria, abdominal pain or distension, nausea with or without vomiting, and fever [5, 6].

Ureteral strictures leading to obstruction can be uncomplicated, or complicated by urinary tract infection, renal insufficiency, or renal failure. Complicated obstruction often requires decompression of the urinary tract with either placement of an indwelling ureteral stent or a percutaneous nephrostomy tube. Ureteral stents are placed, exchanged and removed with a cystourethroscope. For patients requiring a chronic indwelling stent, stents are usually exchanged every three months.As a consequence, bacterial colonization of the ureteral stent, that can lead to severe urosepsis, is common. Other serious complications include hematuria, stent migration, and stent encrustation [7].

In cases of complex ureteral stricture disease were ureteroneocystostomy is not an option, renal transplantation should be considered. Previous small case series have reported minimal complications in this specific group of patients with no evidence of recurrent ureteral strictures [8]. Nephrectomy should never be performed without exploring the option of autotransplantation if there is a reasonable portion of viable ureter.
